# In situ structure of virus capsids within cell nuclei by correlative light and cryo-electron tomography

**DOI:** 10.1038/s41598-020-74104-x

**Published:** 2020-10-19

**Authors:** Swetha Vijayakrishnan, Marion McElwee, Colin Loney, Frazer Rixon, David Bhella

**Affiliations:** MRC-University of Glasgow Centre for Virus Research, Sir Michael Stoker Building, Garscube Campus, 464 Bearsden Road, Glasgow, G61 1QH Scotland, UK

**Keywords:** Biological fluorescence, Viral infection, Cellular microbiology, Herpes virus, Molecular biophysics, Fluorescence imaging, Molecular imaging, 3-D reconstruction

## Abstract

Cryo electron microscopy (cryo-EM), a key method for structure determination involves imaging purified material embedded in vitreous ice. Images are then computationally processed to obtain three-dimensional structures approaching atomic resolution. There is increasing interest in extending structural studies by cryo-EM into the cell, where biological structures and processes may be imaged in context. The limited penetrating power of electrons prevents imaging of thick specimens (> 500 nm) however. Cryo-sectioning methods employed to overcome this are technically challenging, subject to artefacts or involve specialised and costly equipment. Here we describe the first structure of herpesvirus capsids determined by sub-tomogram averaging from nuclei of eukaryotic cells, achieved by cryo-electron tomography (cryo-ET) of re-vitrified cell sections prepared using the Tokuyasu method. Our reconstructions confirm that the capsid associated tegument complex is present on capsids prior to nuclear egress. We demonstrate that this method is suited to both 3D structure determination and correlative light/electron microscopy, thus expanding the scope of cryogenic cellular imaging.

## Introduction

Three-dimensional (3D) information on protein structure is key to our understanding of dynamic processes that occur within a cell or tissue at the molecular level. Electron microscopy (EM) has been a critical tool in the study of cellular ultrastructure. Routine classical methods such as epoxy resin embedding have been employed for imaging of biological material in the electron microscope since the 1950’s^1^. More recently high pressure freezing coupled with freeze substitution (HPF/FS) has been instrumental in improving the quality of biological samples for cellular and tissue EM imaging^2^. However they both involve the use of metal contrasting agents and dehydration of the sample^3,4^. Such methods limit the preservation and visualisation of fine features. Cryo-EM has recently emerged as a powerful tool in the study of high-resolution macromolecular structure, transforming the field of structural biology. Purified protein assemblies are embedded in vitreous ice yielding frozen-hydrated specimens that are suited to imaging in the transmission electron microscope. This method produces artefact-free, high-resolution images of biological assemblies in near-native conditions that may be processed to determine high-resolution structures. These studies have however largely been limited to single-particle analysis (SPA)—the study of purified homogeneous specimens, which may be computationally averaged to yield high-resolution structure data^5^. Many of the most interesting biological questions involve unique and heterogeneous structures that are not amenable to structural averaging methods employed in cryo-EM SPA techniques. To address this challenge, considerable efforts have been made in the development of cryo electron tomography (Cryo-ET) as a tool to image heterogeneous assemblies such as enveloped viruses as well as vitrified cells^6–8^. Owing to the limited penetrating power of electrons, cryo-ET is restricted to samples that are less than 500 nm thick, thus for cellular imaging, only the thin edges of cells are accessible. Features in the interior of eukaryotic cells, such as the nucleus, are not readily viewed. To image the cell interior requires thinning of the area of interest to less than 500 nm. Early attempts to address this problem employed cryogenic microtomy or ‘cryo-sectioning’ of vitreous frozen-hydrated cells. While cryo electron microscopy of vitrified sections (CEMOVIS) avoids chemical fixation and staining, it is technically challenging, limited in sample thickness and subject to several artefacts including compression and crevasses. These restrict the potential of CEMOVIS for high-resolution structural imaging^9,10^. More recently progress has been made towards artefact-free cell sectioning through milling of cellular specimens into electron-transparent lamellae using cryo focused ion beam scanning electron microscopy (cryoFIBSEM)^11^. When successfully applied cryoFIBSEM provides well preserved views of the cell interior. Focussed ion-beam milling of vitreous specimens is, however, a low throughput method that is prone to failure—lamellae are easily broken or contaminated by frost during transfer from the cryoFIBSEM to the cryo-TEM. Moreover, the method involves specialised and expensive equipment.

Artefact-free or low-artefact cryo-microtomy approaches to preparing cellular material for cryo-ET are therefore desirable. Such approaches have the benefit of using less expensive and more widely available equipment for high-resolution 3D imaging of cells and tissues, potentially leading to in situ structure determination. Vitrified frozen cell sections (VFS) provide an ideal compromise wherein chemically fixed samples are cut under cryogenic conditions, thawed and then re-vitrified by plunge-freezing for imaging by cryo-EM or cryo-ET^12,13^. This technique has several advantages for investigators interested in determining macromolecular structures in situ. Cryo-microtomes are already available in many EM laboratories, or existing microtomes may be modified for low-temperature microtomy. The method is relatively high-throughput; many sections may be prepared at once, with a greater likelihood of successful imaging. To our knowledge however, use of VFS has thus far been confined only to imaging of cells and tissue specimens^13–16^. In situ 3D structure determination by means of sub-tomogram averaging has not been hitherto attempted.

While cryo-ET enables imaging of macromolecular structures in 3D within intact cells, it remains challenging to identify features of interest within the crowded and complex environment of the cell. To overcome this problem, methods have been developed that allow investigators to precisely locate features of interest prior to recording tomograms. This is achieved through fluorescence microscopy of cell sections prior to electron imaging and is termed correlative light and electron microscopy (CLEM)^17^.

Here we present a modified strategy for determining in situ 3D structures from re-vitrified cell sections using subtomogram averaging, coupled with CLEM and cryo-ET. We demonstrate the feasibility of this technique by determining structures of the herpes simplex virus type 1 (HSV-1) capsid, imaged within the nucleus of intact infected cells.

HSV-1 is an important human pathogen that causes cold sores. Infection with herpesviruses leads to life-long infection owing to their capacity to enter a latent state^18^. Virion morphogenesis begins in the nucleus of infected cells with assembly of an icosahedral procapsid. Viral genomic DNA is then pumped into the procapsid through a portal assembly located at a unique five-fold vertex^19^. The resulting mature nucleocapsid then buds into the cytoplasm, where it is surrounded by a proteinaceous layer known as the tegument, before budding into plasma membrane derived endocytic membranes; giving rise to mature enveloped virions^20,21^. The nucleocapsid structure has been extensively studied and was recently characterised at high resolution, leading to an atomistic model of this large (1250 Å diameter) and complex assembly^22^. Of particular relevance to this study is the definition of the structure and composition of an assembly variously known as the capsid-vertex specific component (CVSC) or capsid associated tegument complex (CATC). Comprised of three proteins (pUL17, pUL25 and pUL36), this assembly forms a pentaskelion shaped structure that sits over the five-fold symmetry axes^23–25^. The inner tegument protein pUL36 is understood to be the primary bridge between the capsid and tegument layers. The precise site of pUL36 addition to the capsids, in the nucleus or in the cytoplasm, has been the subject of much debate, with the balance of evidence suggesting that it binds capsids in the cytoplasm^26,27^. This is at odds with structural data that show two copies of pUL36 intimately associated with pUL17 and pUL25 to form a 5-helix bundle in the CVSC^22–25^. pUL17 and pUL25 are definitively added in the nucleus. Our data confirm the presence of the CVSC pentaskelion on HSV nucleocapsids in the nucleus, supporting the argument that capsids may bind the tegument protein pUL36 (VP1/2) prior to nuclear egress.

## Results

### Correlative imaging of re-vitrified sections shows well-preserved ultrastructure

To image viral infectious processes within the cell, we used the vitreous frozen section (VFS) method. Virus infected cells were aldehyde fixed prior to being scraped and pelleted. The cell pellet was embedded in gelatin and cut into small cubes that were infused with sucrose. Embedded cells were then frozen in liquid nitrogen and ~ 200 nm thick sections were cut at low temperature (-120ºC). Frozen sections were collected on holey carbon film TEM grids and then washed to both warm the sections and remove the sucrose. Sections were then imaged at room temperature in the confocal microscope to identify regions of interest (ROIs), prior to being re-frozen by plunging into liquid ethane.

**Figure 1:**
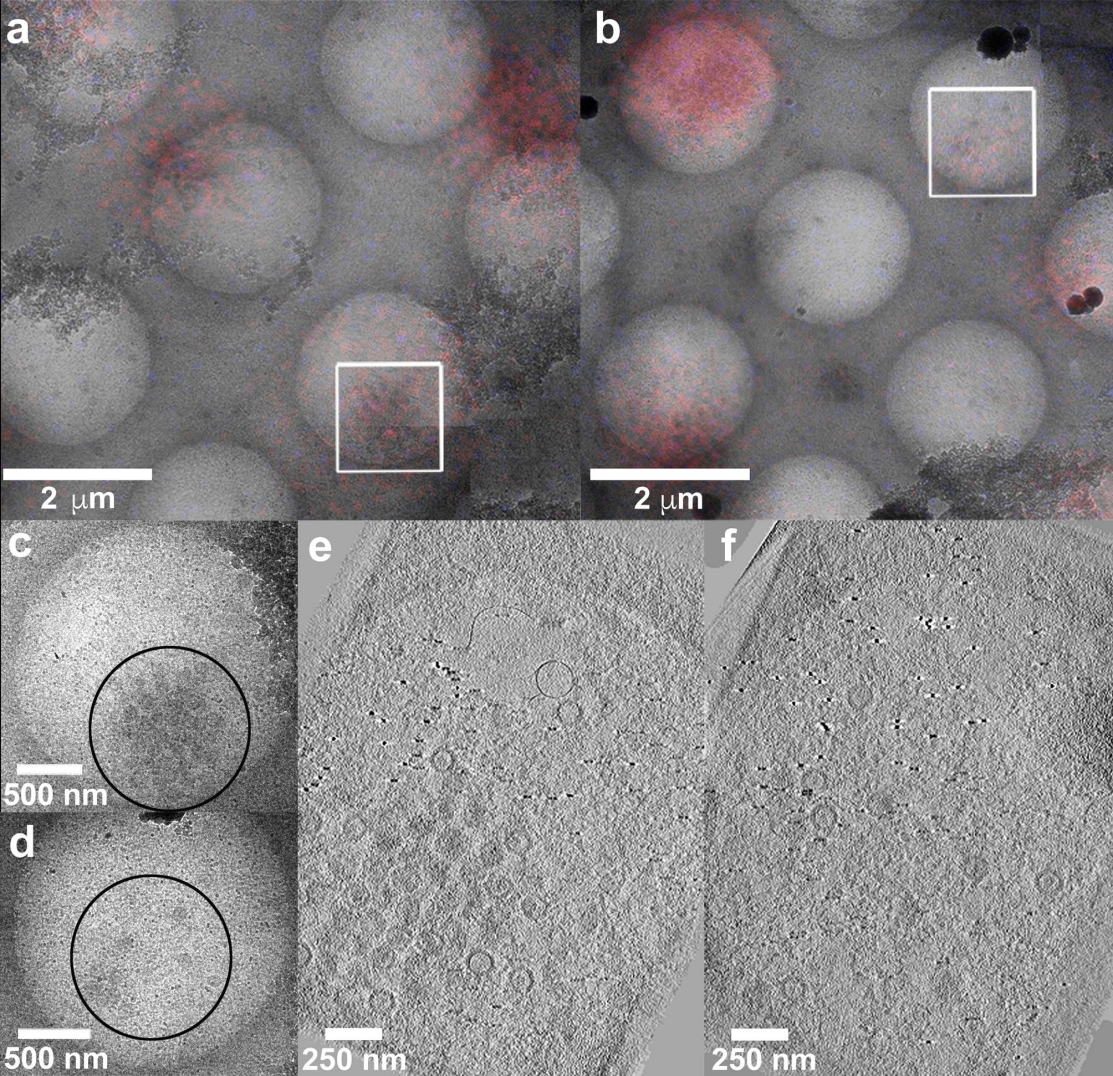
Correlative imaging of HSV virions. (**a**) and (**b**) Medium magnification light microscopy and EM images overlaid to find suitable ROI (white squares) with HSV capsids inside the cell. Wild type HSV-RFP tagged virions are shown (red). (**c**) High magnification EM image of the area of interest (white square in **a**) showing HSV capsids within the cytoplasm. (**d**) High magnification EM image of the area of interest (white square in **b**) denoting HSV capsids in the nucleus. (**e**) and (**f**) Z-slices through tomograms of cytoplasmic and nuclear capsids of areas denoted in (**c**) and (**d**). Appropriate scale bars are shown.

Fluorescence light microscopy (FLM) was used to identify cells containing fluorescently tagged (RFP) wild type HSV-1 capsids, which were located in both the nucleus and cytoplasm. Sections were initially imaged in the laser-scanning confocal fluorescence microscope using tile mode to locate all sections on the grids (Supplementary Fig. S1). Specific sections were chosen for further imaging in differential interference contrast (DiC) and fluorescence modes (RFP, DAPI) to obtain precise positions for HSV-1 capsids within cells (Supplementary Fig. S1a). Sections were then re-vitrified and imaged by cryo-EM and cryo-ET. Initially the entire grid was imaged at low-magnification to detect the ROIs previously identified by light microscopy. We carried out image registration by overlaying the FLM and cryo-EM images initially at low magnifications (100×) to determine precise locations of cells for subsequent cryo-ET (Supplementary Fig. S1b–d). Overlay was then carried out cautiously with FLM (RFP) and cryo-EM images at intermediate magnifications (6000×) to locate ROI within the cell, aiding to discern capsids present in the nucleus and cytoplasm (Fig. 1a,b, Supplementary Fig. S1e,f). Specifically at high cryo-EM magnifications (10,000×), nuclear regions were easily identifiable owing to their characteristic speckled texture that we ascribe to chromatin, visualisation of the nuclear membrane and the presence of well-dispersed capsids. Cryo-EM of the selected ROIs confirmed the presence of many HSV-1 capsids in the nucleus (Fig. 1d,f; Supplementary Fig. S1e) as well as in the cytoplasm (Fig. 1c,e; Supplementary Fig. S1e) of infected cells. Areas for cryo-ET were selected based on the presence of many capsids and minimal frost contamination and tomograms were recorded of capsids within the cytoplasm and the nucleus (Supplemental Video 4). Our images therefore not only indicate good preservation of ultrastructure but also demonstrate the feasibility of using CLEM on re-vitrified sections to identify macromolecules of interest within the dense complex environment of the cell.

### Cellular and capsid 3D ultrastructure revealed by cryo-ET of re-vitrified sections

The inner tegument protein pUL36 of HSV-1 plays a vital role in virion assembly, however the site of its recruitment to the capsid, within the nucleus or the cytosol is unclear and has been a topic of much debate. To investigate this, we imaged refrozen vitreous sections of cells infected with the non-fluorescently tagged mutant FRΔUL37—capsids that lack the pUL37 tegument protein but have pUL36. Cryo-EM images showed excellent preservation of cell ultrastructure combined with negligible cutting artefacts.

Although Tokuyasu sections are prone to cutting artefacts such as compression during sectioning at cryogenic temperatures, the elastic properties of the material when floated on water restores the sections to a stretched and uncompressed state^10,28^. Further, the use of chemical fixatives and sucrose in these sections prevents any damage upon thawing when floated on water^14^. No obvious compression, knife marks or ellipsoidal shaped capsids were observed in sections. Diameter measurements of about 20 randomly oriented capsids over an angular range of 0–360° indicated low standard deviation (SD) in the overall mean diameter at each angle, from the expected capsid diameter (125 nm, Supplementary Fig. S2). Further, variation in mean diameter was within the SD error estimates and showed a fairly flat trend, indicating that the sections exhibit negligible compression (Supplementary Fig. S2b).

**Figure 2:**
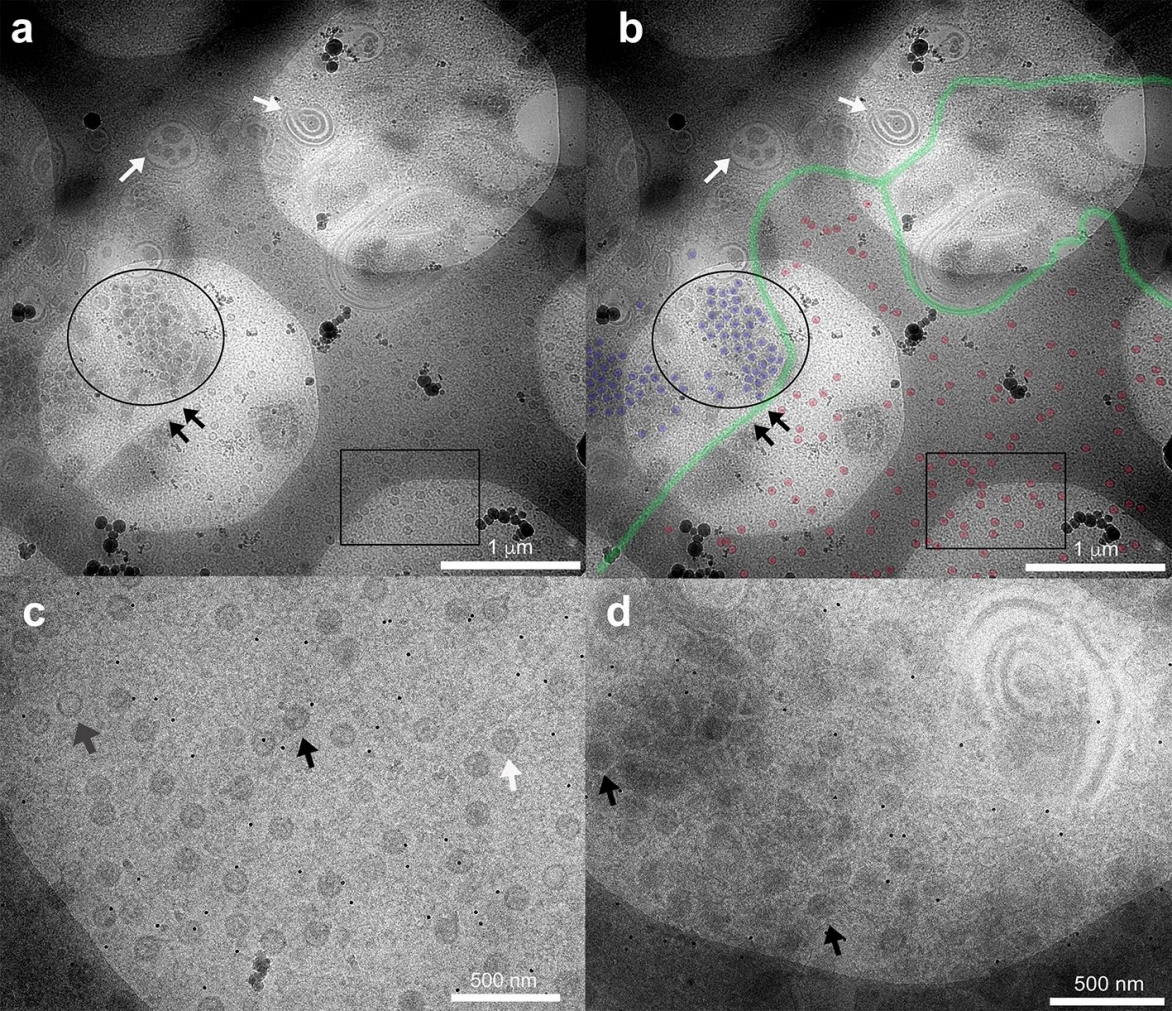
EM imaging of refrozen cell sections. (**a**) Low magnification diffraction imaging of untagged pUL37-null HSV mutant infected cell section by EM. Frozen sections show well-preserved structure and contrast. While an abundance of HSV capsids within the nucleus (black rectangle) and cytoplasm (black circle) are seen separated by the nuclear membrane (black arrows), other membranous and vacuolar structures (white arrows) can also be observed within the dense cytoplasm. (**b**) For easy visualisation, segmentation of the image in (**a**) using Photoshop is shown annotating probable edges of the nuclear membrane (green), nuclear capsids (red) and cytoplasmic capsids (blue). High magnification EM images of (**c**) nuclear and (**d**) cytoplasmic capsids clearly denote uniform dispersal of capsids within the nucleus, and a greater tendency to cluster up within the densely packed cytoplasm. The three different types of capsids (in **c** and **d**) namely, A-type (empty capsids, grey arrow), B-type (scaffold-containing capsids, white arrow) and C-type (DNA-containing capsids, black arrow) have been annotated within the nucleus and cytoplasm. Appropriate scale bars are shown for the different images.

**Figure 3:**
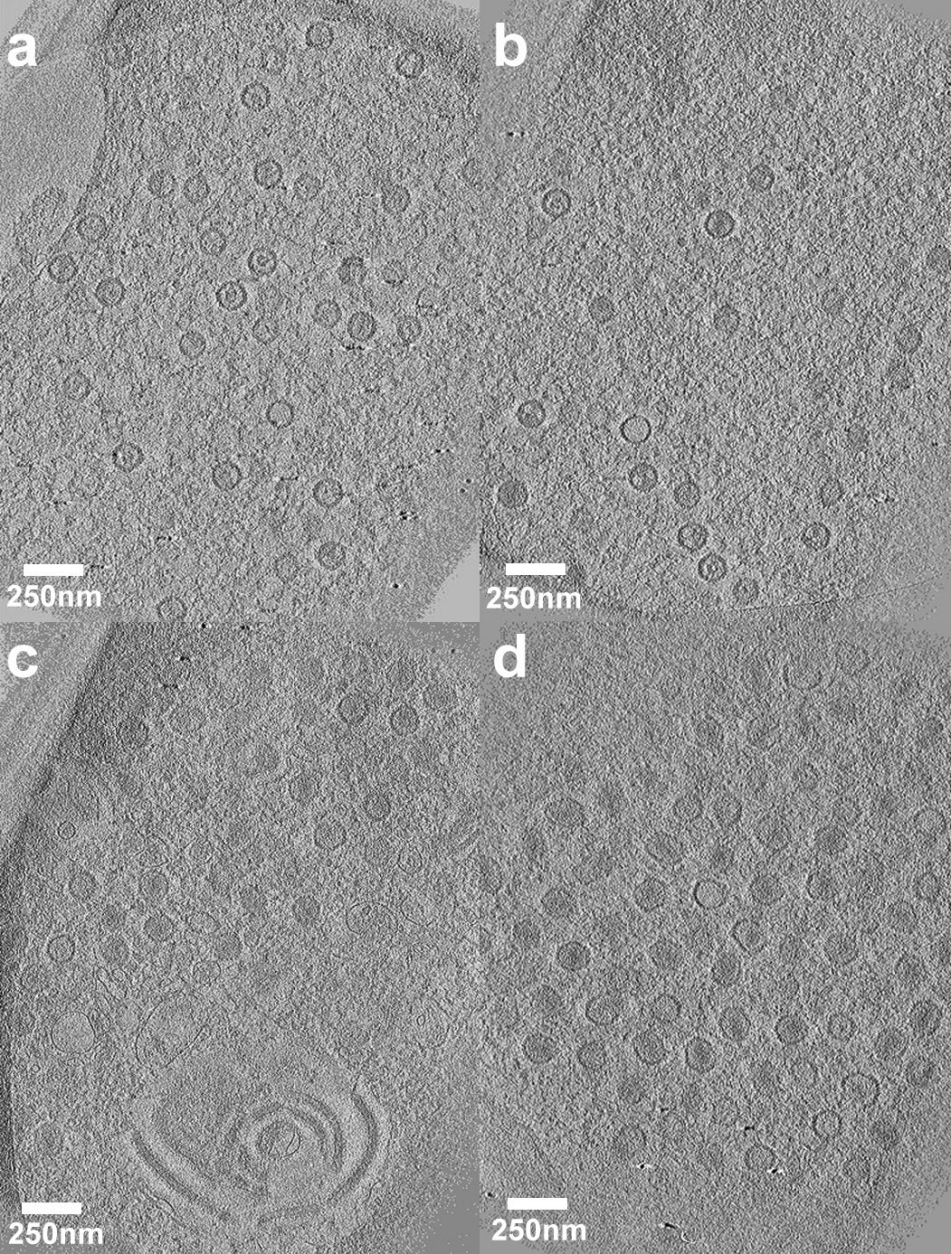
Electron tomography of nuclear and cytoplasmic HSV capsids. Tomograms were collected of the untagged pUL37-null mutant (FRΔUL37) capsids from within the nucleus and cytoplasm. While (**a**) and (**b**) represents a slice along the z-axis of the tomograms that show well dispersed areas of capsids within the nucleus, (**c**) and (**d**) are slices through tomograms showing capsid accumulation in the tightly-packed dense environment of the cytoplasm along with other membrane structures. Capsids in (**c**) and (**d**) look deformed, likely due to the phenotype of significant aggregation exhibited by the pUL37-null mutant. Scale bar = 250 nm.

At low magnification the nuclear membrane was seen demarcating the nucleus from the cytoplasm (Fig. 2a,b). Several membranous and vacuolar structures were also visible within the cytoplasm. Cellular organelles such as mitochondria were observed and appeared to be well preserved (data not shown). It is noteworthy that while low magnification and high contrast provides an overall view of the cell, the nuclear membrane was occasionally unclear (Fig. 2b). This is in contrast to imaging at high magnifications where we saw the nuclear membrane clearly alongside an abundance of well distributed capsids within the speckled texture of the nucleus. In the cytoplasm the mutant capsids were found to be concentrated in dense aggregates as a result of the lack of pUL37 (Fig. 2c,d). The pUL37 mutant capsids have been shown previously to significantly aggregate in the cytosol, implying the protein’s critical role in the release of mature virions from infected cells^29^. The majority of cytosolic capsids in these accumulations look deformed, with few appearing intact (Fig. 3). This deformation is likely to be a result of the extensive aggregation exhibited by this mutant rather than a compression artefact in the sections, which appears to be negligible (Supplementary Fig. S2). It is also noteworthy that a compression would likely show deformation of capsids in a uniform direction a phenomenon that we do not observe in our sections. At higher magnification, the different types of capsids within the nucleus were easily discerned; empty A-capsids, scaffold-protein containing B-capsids and DNA filled C-capsids (Figs. 2, 3). We performed cryo-ET on capsid-rich areas within the nucleus and cytoplasm to generate 3D structure data. As expected, cryo-ET of capsids within the nucleus revealed an abundance of B- and C- capsids (Fig. 3a,b, Supplemental Video 5). Capsids in the cytoplasm were closely packed and primarily C-capsids (Fig. 3c,d).

### 3D structure of the intranuclear HSV capsids by subtomogram averaging

To establish whether tomograms of refrozen cell section material are sufficient to determine 3D structures by sub-tomogram averaging, we sought to calculate *in-situ* reconstructions of HSV-1 capsids.

**Figure 4:**
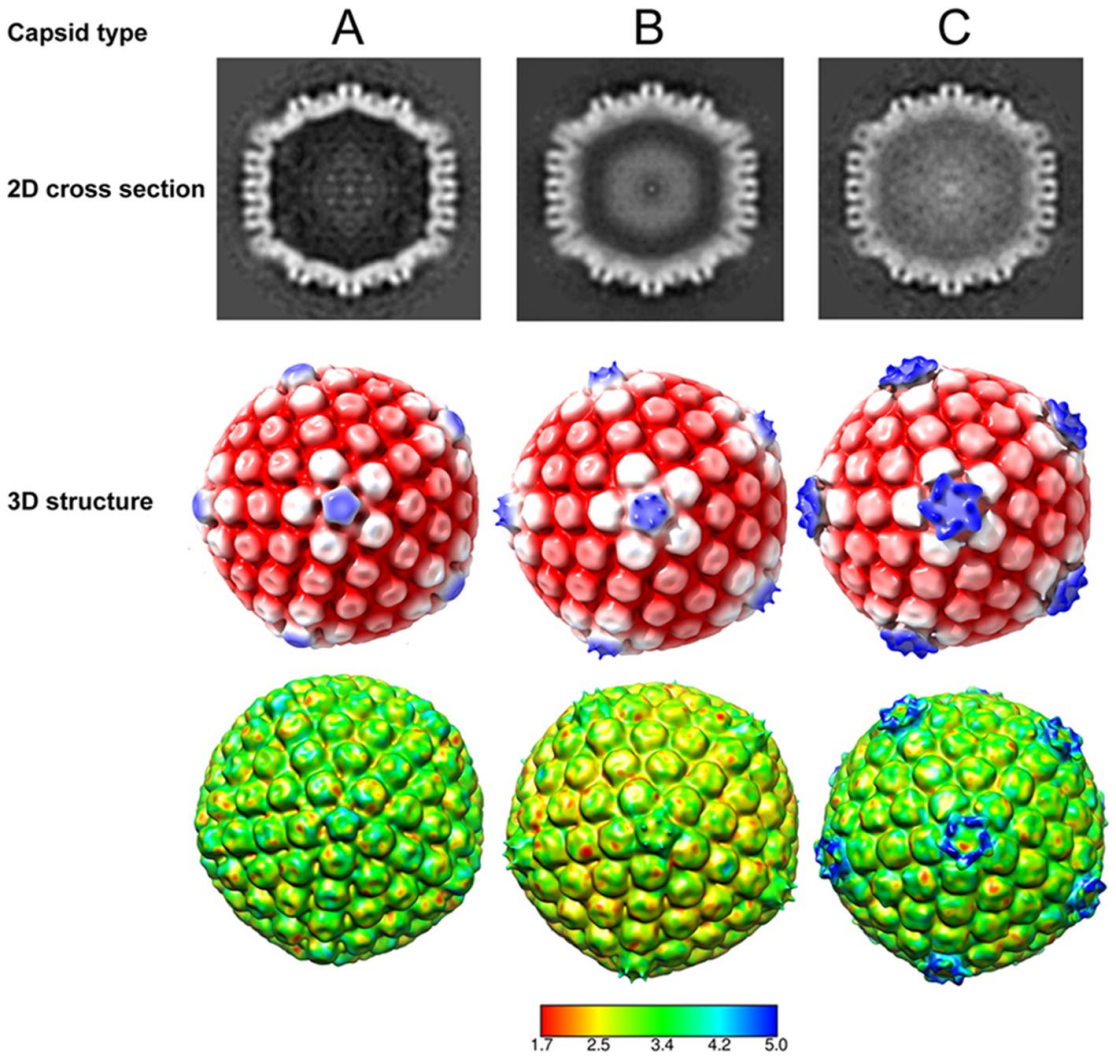
Determination of the 3D HSV capsid structures from within the nucleus. 3D structures of the three types of nuclear capsids, A-, B- and C-type were determined by subtomogram averaging. 2D cross-section images clearly indicate density differences in the interior of the capsid between the three types. Density relating to scaffolding proteins and DNA is seen inside the capsids within the B-type and C-type, respectively, while none is present inside the empty capsid shell of the A-type. The 3D structures obtained have been radially coloured according to distance (row 2) and local resolution estimates (row 3, colour key in nm) at low thresholds of density and are shown along the fivefold axis of icosahedral symmetry. Pronounced pentaskelion density is observed above the pentons only in the C-type capsids.

The inner tegument proteins pUL36 and pUL37 of HSV-1 play a critical role in virion assembly^29–31^. There has been uncertainty in the HSV field regarding the point in the virion morphogenetic pathway at which pUL36 and pUL37 are recruited to the capsid, if this happens within the nucleus or after nuclear egress^26, 27^. To shed light on this question, we carried out cryo-ET on the mutant lacking pUL37 (FRΔUL37). As capsids within the nucleus were discrete and well separated (Fig. 3a,b), we performed subtomogram averaging to determine the 3D structures of the 3 classes of capsid present, (A, B and C-capsids). In total 97 A-capsids 526 B-capsids and 155 C-capsids were selected and processed by sub-tomogram averaging. Following several rounds of 3D classification and refinement along with imposition of icosahedral symmetry, reconstructions were calculated to a resolution of 5.7 nm (A-capsids), 5.6 nm (B-capsids) and 6.4 nm (C-capsids) for each class as validated by the Fourier shell correlation (FSC) assessments (Supplementary Fig. S3). Cross-sections of the structures obtained for each of the capsid classes showed clear differences in density between them; with A capsids being empty, while the B and C capsids harboured density within, corresponding to scaffold protein and DNA, respectively (Fig. 4). Inspection of the capsid reconstructions revealed differences in CVSC density observed at the fivefold vertices between the three subclasses; the pentaskelion structure is clearly visible in the C-capsids (Fig. 4, Supplemental Video 6). Local resolution analysis revealed that much of the capsid was resolved at 3.5 nm, while only the density at the fivefold vertices was less well resolved beyond 4.5 nm (Fig. 4).

**Figure 5:**
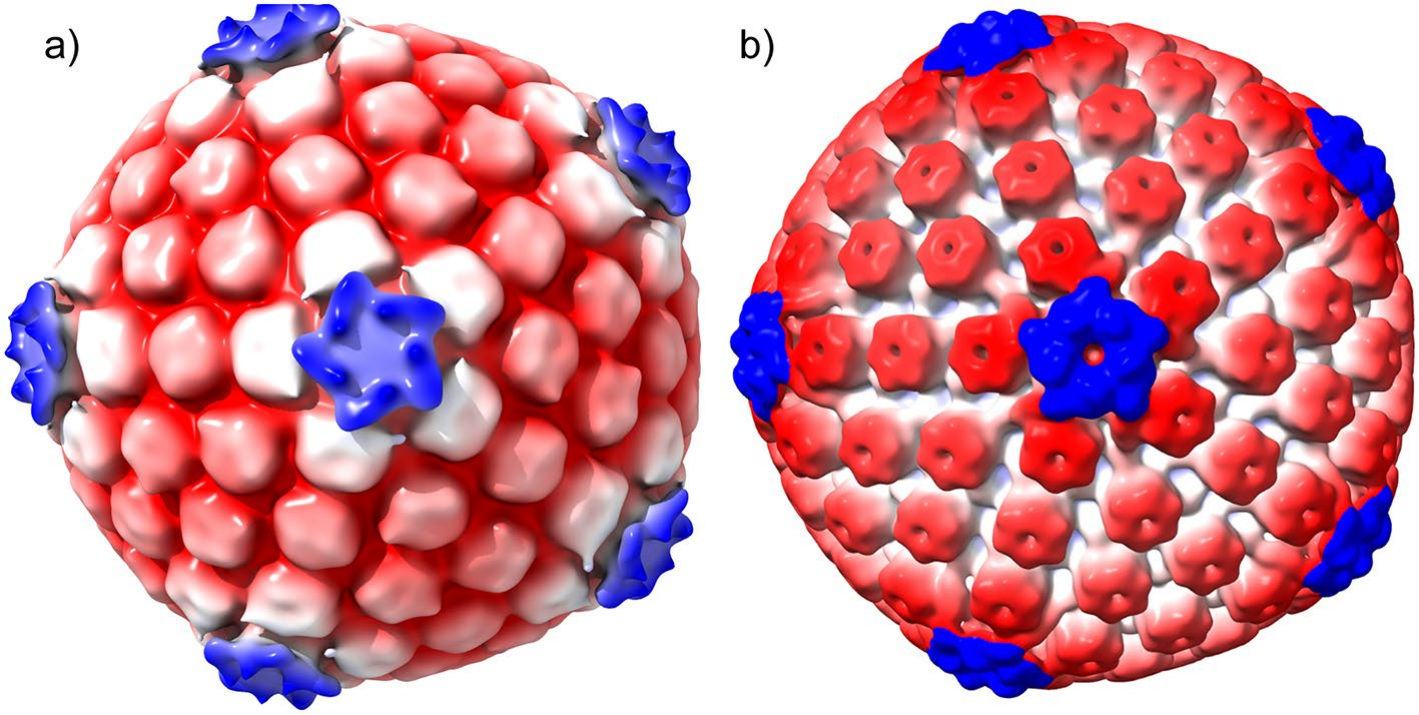
Comparison of in situ intranuclear capsid structure with the structure of virion nucleocapsid. (**a**) The in situ 3D structure of C-capsid determined by subtomogram averaging at 6.4 nm shows clear pentaskelion electron density above the pentons at low thresholds of density. This corresponds to the CVSC, reported to be composed of pUL36, pUL17 and pUL25 proteins; and is reasonably similar to the (**b**) structure of purified virion nucleocapsid at a comparable resolution^19^. Capsids are radially coloured from the centre.

In recent high-resolution single particle reconstructions of capsids within purified HSV-1 virions, the pentaskelion structure can be clearly seen above the penton vertices and has been attributed to the proteins pUL17, pUL25 and pUL36^19,22^. It has also been previously shown at intermediate resolutions that nuclear capsids containing pUL36 and not pUL37 show a strongly resolved star-like density compared to mutants where pUL36 is absent, suggesting the presence of pUL36 in the CVSC density^24^. Although at low-resolution, the star-shaped pentaskelion density observed in our C-capsid structure is strongly reminiscent of the penton density in these intermediate and high-resolution structures determined from purified HSV-1 virions and mutants at the equivalent resolution (6.4 nm, Fig. 5). While the CVSC star-like density is readily seen over the pentons in the C-capsid, the density extending from the penton to the triplexes is less clear (Fig. 5a) than in the virion nucleocapsid (Fig. 5b). We attribute this to the low particle numbers used in our reconstruction. Moreover, at this resolution (> 5 nm) the penton density observed is not sufficiently well resolved to allow us to ascertain the composition of the CVSC in our C-capsid structure.

The CVSC density was hardly visible in the B-capsids. We attribute this to low occupancy of CVSC in this class of capsids, resulting in its signal being lost during particle and symmetric averaging. The CVSC density is completely absent in the A-capsids.

## Discussion

Cryo-EM is fast becoming a critical tool for structural biologists, allowing atomic-resolution structures of proteins and viruses to be calculated rapidly^5^. In this work, we have addressed the considerable challenge of obtaining 3D structures of macromolecular assemblies from within the native context of the cell under cryo conditions. By adapting the method of classical Tokuyasu sectioning coupled with CLEM and subtomogram averaging, we offer an easy and attractive method for imaging and 3D structure determination of proteins/viruses from within cells^12^. The Tokuyasu method has been previously used to produce sections that were subsequently vitrified for cryo-EM imaging^13^. However, the use of vitrified frozen sections (VFS) has thus far been limited only to imaging of cells and tissue sections by cryo-EM and cryo-ET, and not to 3D in situ structural determination^13–15^.

We believe these methods offer a less technically challenging and relatively inexpensive alternative to FIB-SEM, that would allow the majority of cryo-EM laboratories to carry out 3D structural characterisation of macromolecular assemblies in cellular and tissue samples. Further, the option of using CLEM on these sections offers the great advantage of precisely locating sub-cellular structures of interest, broadening the applicability of this method to the study of low-frequency events. 3D structure determination of macromolecular assemblies from re-frozen sections via subtomogram averaging provides a starting point to more routine high-resolution structure determination of proteins from within the cell.

Aldehyde fixation has been successfully used in solving protein and virus structures by cryo-EM at intermediate resolutions^32–34^. However, as fixation in a cellular environment proceeds differently to that in vitro, it may cause some structural artefacts, possibly contributing to the low resolution of capsid structures in our study (5–6 nm), in comparison to resolutions obtained from subtomogram averaging of proteins from unfixed cryo-ET of for example purified virions (0.8–2 nm). In the absence of comparable data from FIB-SEM prepared nuclear HSV capsids, it is not possible to directly compare achievable results and thereby unambiguously assign fixation for the low resolution achieved here. We think that a crucial factor limiting resolution in our data is likely to be insufficient particle numbers. Although the current structures are at low resolution, our method serves as a signpost for further improvement to achieve higher resolutions. Carrying out automated data collection on a high voltage (300 kV) microscope with high-speed direct electron detectors will result in increased particle numbers, potentially allowing structure determination to intermediate-to-high resolution.

In this study we chose the highly symmetric and well characterized HSV capsid as a model to test the feasibility of our method to determine the in situ 3D structures of intracellular capsids by subtomogram averaging. Employing a correlative approach using CLEM, allowed us to spatially locate capsids within both the nucleus and cytoplasm. Well-preserved fluorescence signal as well as ultrastructure within the sections observed was consistent with previously published work describing correlative imaging of refrozen Tokuyasu sections^16^.

A complex variable proteinaceous tegument layer, comprising more than 20 proteins surrounds the HSV capsid. Of these, pUL36 and pUL37 are classified as ‘inner-tegument’ proteins and are tightly associated with the capsid. The precise location of pUL37 has not been determined. pUL36 is intimately associated with pUL25 and pUL17 to form the CVSC at the five-fold vertices^35–37^. With the pUL37-null mutant used in this work, accumulation of C-capsids in dense clusters was observed in the cytoplasm of infected cells, confirming that the tegument protein pUL37 is not important for capsid assembly, DNA packaging or nuclear egress, but plays a vital role in tegument addition within the cytoplasm leading to envelopment. Despite extensive studies on HSV-1 capsid morphogenesis, controversy persists concerning the cellular compartment in which the tegument protein pUL36 is added to the nascent virus capsid. The CVSC density observed at capsid five-fold vertices in cryo-EM studies of mature virions, includes a five-helix bundle that is the principal site of interaction for two copies each of pUL36 and pUL25 along with a single copy of pUL17^26,35,37–40^. The intranuclear capsid structures obtained from subtomogram averaging here clearly indicate variable pentaskelion CVSC density over the pentons between the three subgroups of capsids; B-capsids having weak density compared to C-capsids, likely a consequence of low occupancy of CVSC in B-capsids. Moreover, our analysis revealed star-like CVSC density at the penton vertex in the C-capsids that is comparable, albeit at low-resolution, to previously reported high-resolution structures of capsids within purified HSV-1 virions^19,22^. Although the presence of putative pUL36 in the pentaskelion density of these low-resolution capsid structures cannot be confirmed, our data strongly argue for a fully assembled CVSC prior to nuclear egress. We cannot rule out the proposed incorporation of N-terminally truncated forms of pUL36 however^41–43^.

In summary, we have demonstrated a relatively inexpensive and technically straight-forward method for determining in situ 3D structures of macromolecular assemblies using CLEM and subtomogram averaging. Despite the wide applicability of this method, it could be improved and optimised for the future; automatic data collection of large datasets using 300 kV microscopes with high-speed direct electron detectors would be an obvious starting step. This may enable determination of intermediate-to- high-resolution structures from re-vitrified sections of cells and tissues by subtomogram averaging techniques. Finally, we validated this method by determining the first structures of icosahedral capsids directly determined from within the nucleus. Our method opens the possibility of determining and characterising specific complexes and their interactions at low-to-intermediate resolution within the functional context of the cell or tissue, providing snapshots of important and dynamic events in biology.

## Materials and methods

### Cells and viruses

Baby hamster kidney cells (BHK-21) were cultured as described previously^24,29^. Briefly, cells were grown at 37 °C in Glasgow minimum essential medium (GMEM; Invitrogen) supplemented with 10% newborn calf serum (NCS; Invitrogen) and tryptose phosphate broth (TPD; Invitrogen). BHK-21 cells were infected using wild-type HSV-1 (strain 17^+^) pUL35 RFP or UL37-null (FRΔUL37) virus at 10 PFU per cell and incubated for fixed times post infection.

### Preparation of HSV infected cells for sectioning

Petri dishes (60/100 mm) dishes were cultured with BHK-21 cells and grown to confluency prior to infection with wild type pUL35RFP1D1 (pUL35 tagged with the RFP fluorophore) or FRΔUL37 strains of HSV at 10 PFU/cell for 12 h and 9 h post infection, respectively. Cells were then washed with PBS and fixed with 2% formaldehyde and 1% glutaraldehyde in PBS for 5 h at room temperature or at 4 °C overnight. The following day, cells were washed with PBS, scraped, pelleted and resuspended in 12% porcine gelatin (Sigma) and cooled on ice. Cooled pellets were cut into 1 mm cubes and infiltrated with 2.3 M sucrose at 4 °C for 2–3 days. Well-infiltrated blocks were attached to Tokuyasu stubs (Leica Microsystems, Germany), frozen in liquid nitrogen and stored in cryotubes in liquid nitrogen dewars until future use.

### Tokuyasu sectioning

Sucrose-infiltrated frozen cell blocks were transferred to the cryo ultramicrotome (Ultracut EM FC6, Leica Microsystems). 200–220 nm thick sections were cut with a cryo-immuno diamond knife (Diatome) and were collected using a “Perfect loop” (Diatome) with 2.3 M sucrose^12^ and transferred to holy carbon finder grids (R3.5/1 Copper NH2—Quantifoil, Jena, Germany). Sections were stored at 4 °C until further processing.

### Correlative confocal light microscopy

Cut sections were washed with PBS to remove all traces of sucrose. Prior to imaging, DAPI (4′,6-diamidino-2-phenylindole dihydrochloride, Sigma, 1:500 dilution) was applied for 30 min followed by washes in PBS (3 × 10 s). Sections on the grid were then transferred to glass-bottom dishes (Mattek Corp, USA) on ice with sections facing down submerged in minimal buffer (~ 800 μl) and imaged. Confocal laser scanning microscopy was performed on the LSM 880 microscope (Zeiss, Germany) with a 63x/1.4 NA oil immersion objective. Tiled images (20 × 20 tile) of the entire grid as well as single images and image stacks (2048 × 2048 × 5) were acquired with a pinhole close to 1 Airy unit. DAPI (405 nm excitation) and RFP (530 nm excitation) signals were acquired with GaAsP detectors (Zeiss, Germany). Image processing and deconvolution was carried out with the Zeiss licenced software, ZEN Black.

### Cryo EM/ET and tomogram reconstruction

Sections imaged by light microscopy were re-vitrified and prepared for cryogenic transmission electron microscopy using an FEI Vitrobot Mk IV. Briefly, 6–8 μl of 15 nm colloidal gold (1:3 dilution, BBI, UK) was applied to the finder grids with sections and blotted for 5 s before plunging into liquid ethane^44^. Vitrified samples were viewed at low-temperature (around 98 K) and under low electron dose conditions using a JEOL 2200 FS cryo-microscope operated at 200 kV with zero-loss energy filtered imaging using a slit-width of 30 eV. Samples were held in a Gatan 914 high-tilt cryo-stage. CEM images were typically recorded at zero tilt at low magnifications of 100×–200× as well as medium magnifications of 6000×–8000× using the Gatan Ultrascan 4 k × 4 k CCD camera to determine the same ROIs and enable superposition with light microscopy images. Tilt series were then recorded using the SerialEM software package^45^ at 10,000 × magnification on a Direct-Electron DE20 DDD camera at a sample rate of 5.98 Å/pixel. Images were acquired at two-degree increments with a bi-directional tilt scheme ranging from − 20° to + 60° for the first tilt set followed by − 20° to − 60° for the second tilt set. The target defocus was set between 4–10 m and the electron dose ranged from 50–100 e/Å^2^ per tilt-series. Tomograms were calculated and visualized using the IMOD software package^46^. Reconstruction was performed using weighted back projection followed by CTF correction available as part of the IMOD package.

### Registration of LM and EM images

Alignment of light and electron microscopy images was done with Adobe Photoshop CS5 software. Firstly, the LM image was aligned based on the markings of the finder grid, manually inserted landmarks such as ice contamination as well as RFP tagged HSV and nuclear DAPI signals and their corresponding signals on the cryo-EM image at low magnification (100×). After rough alignment, fine alignment was performed by registering the pattern of holes within the grid square and/or the pattern of cells and their corresponding signals on the cryo-EM image. For easy viewing of ROI, overlay was then carried out carefully with RFP and cryo-EM images at higher magnifications (6000×) to discern capsids present inside the nucleus and cytoplasm. For data presentation, the light and electron microscopy images were merged with the merge channels option in Adobe photoshop CS5.

### Computational subtomogram image processing

Micrograph movies containing 20 frames were processed to calculate a 3D reconstruction using Relion-3.1^47^. Initially movies were corrected for specimen movement and radiation damage using Direct-Electron open source software scripts (DE_combine_references.py and DE_process_frames.py). Processed images were stacked using in-house scripts and tomograms generated with defocus estimation (Ctfplotter) using IMOD^46^. An initial set of 778 particles from 33 tomograms of nuclear capsids was manually picked and extracted using the EMAN program e2boxer.py^48^. Extracted particles were grouped separately based on them being A, B or C-type capsids prior to classification. 3D classification was performed in Relion-3.1 imposing icosahedral symmetry. For the 3D classification, a low resolution starting model of the HSV capsid, obtained after processing and refining ~ 100 manually extracted particles from our tomogram dataset via EMAN2 was used^47,48^. The subvolumes and the starting model were 2 × binned to speed up processing. A subset of particles from each of the three subgroups (A, B, and C-type capsids) was selected from the 3D class that yielded the highest resolution and subjected to further refinement. Global resolution assessment was done with Relion, using the postprocessing task to mask the density maps and calculate the ‘gold-standard’ Fourier shell correlation. Local resolution assessment was then carried out using the local resolution routine in Relion to obtain local resolution estimates across the diameter of the capsid. The final 3D reconstructions of A, B and C-type capsids were visualized in UCSF Chimera, ChimeraX and IMOD^46,49,50^ Movies were created using ChimeraX^50^.

## Supplementary information

Supplementary Figures.

Supplementary Video 4.

Supplementary Video 5.

Supplementary Video 6.
