# Supplementary material for: In situ structure of virus capsids within cell nuclei by correlative light and cryo-electron tomography
Source: Sci Rep. 2020 Oct 19;10:17596. doi: 10.1038/s41598-020-74104-x (PMC7572381; doi:10.1038/s41598-020-74104-x)
Supplement: Supplementary file 1 — Supplementary Figures. [file 41598_2020_74104_MOESM1_ESM.pdf]

In situ structure of virus capsids within cell nuclei by correlative light and cryo-electron tomography.

Swetha Vijayakrishnan, Marion McElwee, Colin Loney, Frazer Rixon, David Bhella

Supplemental data

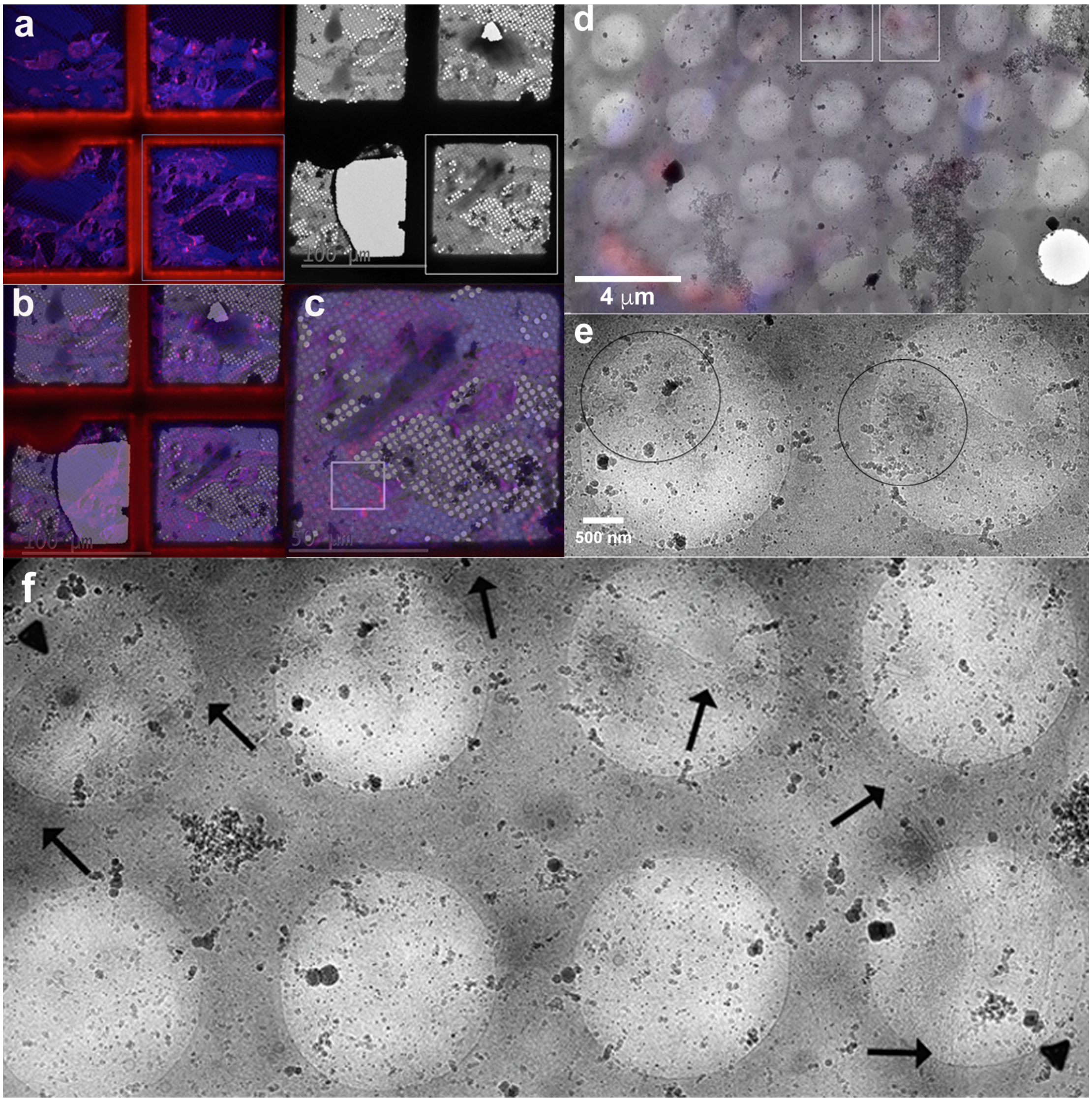

Figure S1. Correlative imaging of HSV capsids.

Overlay of LM and EM images at low magnifications to obtain areas of interest pertaining to wild type HSV capsids in cells, both within the nucleus and cytoplasm. **(a)** On the left is a low magnification confocal image of an area within the cell section depicting RFP tagged HSV (red) infected cells. Blue denotes DAPI staining. The right side is the equivalent area imaged by EM at low magnifications. **(b)** Image registration and overlay of both low-magnification LM and EM images shown in (a). Although the film of the bottom left grid square broke during the freezing process as seen in the EM image (a, right panel image), the remaining intact squares enabled

accurate correlation. **(c)** Close up of the LM-EM overlaid image of the grid square of interest (white square of (a)) showing the chosen position of a cell in greater detail (white square) for further CET analysis **(d)** LM and EM image superposition at medium magnifications of the chosen cell depicted in the white square of (c). High-magnification **(e)** EM images of regions selected in (d) shown as white squares reveal HSV capsids present in both the nucleus and cytoplasm. (f) Correlation with cryo-EM image at intermediate magnification (6000x) helped clearly discern the nuclear (black arrows denoting the nuclear membrane) and cytosolic regions (black triangles, at the top left and bottom right of figure showing the plasma membrane) in the cell and the capsids within them.

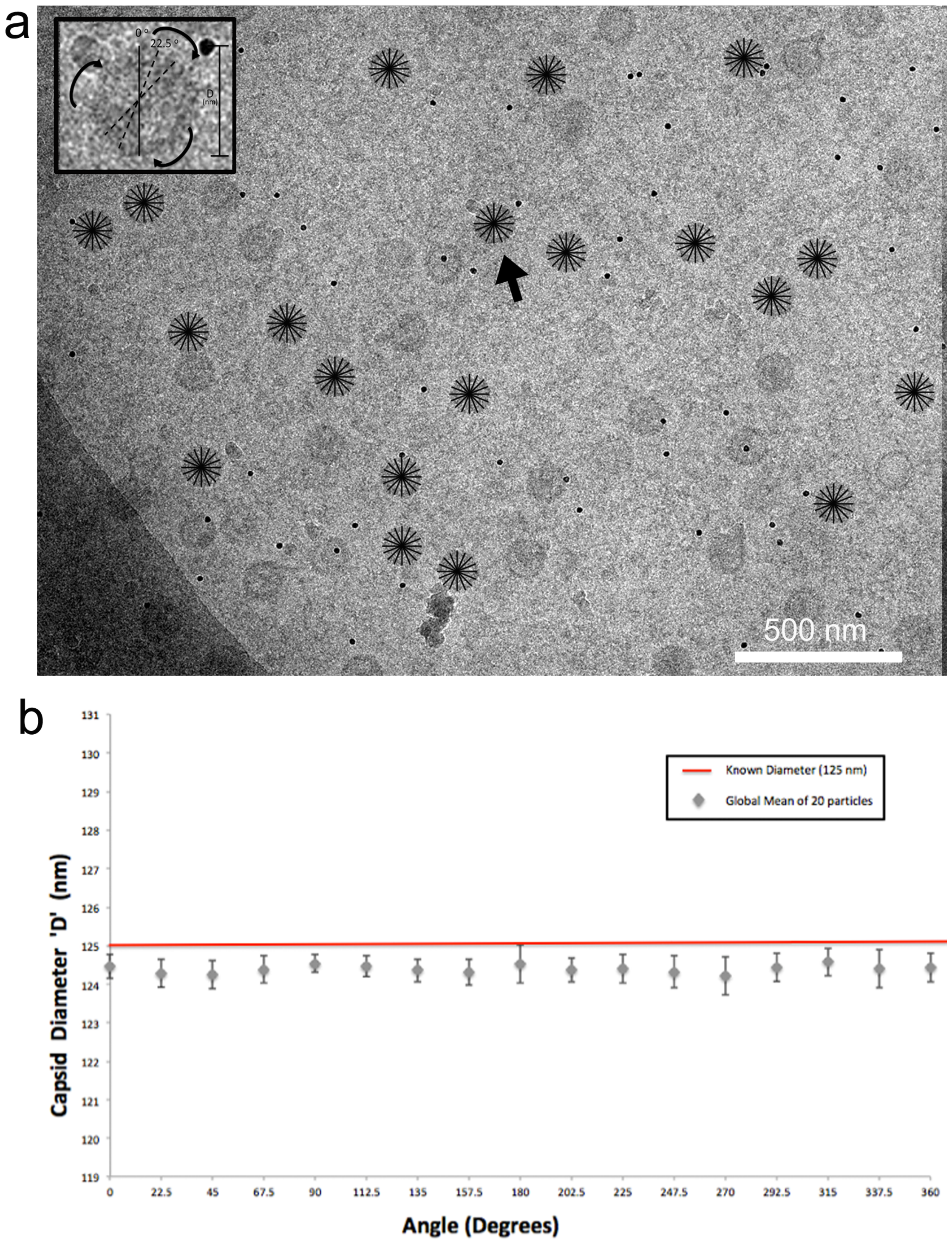

Figure S2. Compression assessment in cryo revitrified-secctions

(a) Assessment for compression in cell sections was carried out by measuring the diameter of HSV capsids in a 2D cryo-EM image over an angular range of $0-360^{\circ}$ and an angular step of 22.5° (inset). (b) Diameter measurement analysis was carried out for

20 capsids and the mean and SD of these over the angular range 0°-360° was calculated and plotted. Variation in diameter of these randomly oriented particles was within the error estimates and depicted a fairly flat trend, thus indicating negligible or nearly no compression. The known capsid diameter (125nm) is also shown (red line).

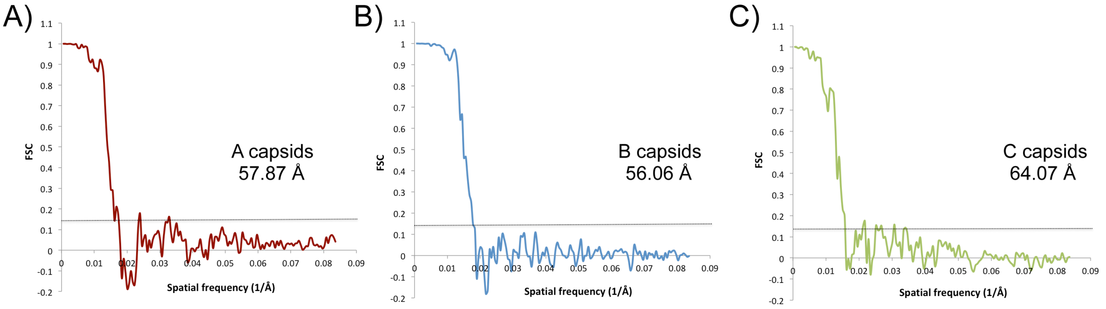

Figure S3. Resolution assessment of HSV capsid structures.

Resolution assessment of 3D reconstructions of virus capsids was determined by the gold standard Fourier shell correlation (FSC) analysis, as part of the Relion-3.1 package. Graphs denoting the resolution of capsids calculated at the FSC 0.143 cutoff to be **(A)** 57.87 Å for A-capsids, **(B)** 56.06 Å for B-capsids and **(C)** 64.07 Å for C-capsids are shown.

Supplemental Video 4. Tomograms of wild type HSV capsid within the cell cytoplasm and nucleus.

Movie showing serial sections through the z-axis of the tomogram of HSV capsids located via correlative imaging within the cytoplasm and the nucleus. Movie created using IMOD and Quicktime.

Supplemental Video 5. Tomograms of UL37-null mutant HSV capsids within the nucleus.

Movies denoting serial sections through tomograms of the UL37 mutant HSV capsids within the nucleus of a cell. Capsids are well dispersed throughout the nucleus and the three different capsids namely, A-, B- and C-type can be seen clearly. Movies were created using IMOD and Quicktime.

Supplemental Video 6. *In situ* 3D reconstructions of intranuclear HSV capsids - A, B and C.

Movies depicting the 3D reconstructions of the subclasses of capsids (A, B and C) within the nucleus obtained by subtomogram averaging. Variation in the additional density over the penton corresponding to the CATC can be clearly seen, with the C-capsids exhibiting the most apparently visible star-shaped density. Movies were created using ChimeraX.
